# Custom-Made Implants in Ankle Bone Loss: A Retrospective Assessment of Reconstruction/Arthrodesis in Sequelae of Septic Non-Union of the Tibial Pilon

**DOI:** 10.3390/medicina58111641

**Published:** 2022-11-14

**Authors:** Silvio Caravelli, Giuseppe Ambrosino, Emanuele Vocale, Marco Di Ponte, Giulia Puccetti, Carlo Perisano, Tommaso Greco, Vito Gaetano Rinaldi, Giulio Maria Marcheggiani Muccioli, Stefano Zaffagnini, Massimiliano Mosca

**Affiliations:** 1 II Clinic of Orthopaedic and Traumatology, IRCCS Istituto Ortopedico Rizzoli, 40136 Bologna, Italy; 2 Orthopaedics and Trauma Surgery Unit, Department of Ageing, Neurosciences, Head-Neck and Orthopaedics Sciences, Fondazione Policlinico Universitario Agostino Gemelli IRCCS, 00168 Roma, Italy

**Keywords:** osteomyelitis, tibia, ankle, custom-made, 3D printing, arthrodesis

## Abstract

*Background and Objectives*: Treating segmental tibial and ankle bone loss after radical surgery for chronic osteomyelitis is one of the most challenging problems encountered by orthopaedic surgeons. Open tibia and ankle fractures occur with an incidence of 3.4 per 100,000 and 1.6 per 100,000, respectively, and there is a high propensity of developing fracture-related infection with associated chronic osteomyelitis in patients. Segmental tibial and ankle bone loss have recently received new and improved treatments. *Materials and Methods*: Above all, 3D printing allows for the customization of implants based on the anatomy of each patient, using a personalized process through the layer-by-layer deposition of materials. *Results*: This article presents different cases from the authors’ experience. Specifically, four patients suffered tibia and ankle fractures and after radical surgery for chronic osteomyelitis combined with high-performance antibiotic therapy underwent ankle reconstruction/arthrodesis with custom-made tibial spacers. *Conclusions*: Thanks to 3D-printed patient-specific devices, it is possible to perform surgical procedures that, for anatomical reasons, would have been impossible otherwise. Moreover, an improvement in overall functionality and an important reduction in pain were shown in the last follow-up in all patients.

## 1. Introduction

Open distal tibia and ankle fractures occur with an incidence of 3.4 per 100,000 and 1.6 per 100,000, respectively, and there is a high propensity of developing injury-related infection with associated chronic osteomyelitis in patients [1,2]. Tibial pylon fractures account for about 1% of all lower limb fractures and 10% of all tibial fractures but can be considered a management challenge for the orthopedic surgeon. This because a significant number of the patients treated develop formidable complications, among which one of the most devastating is infection and nonunion with bone substance loss [3,4].

In cases where there is significant contamination or bacterial load, infection with more virulent or multiple organisms, or complicating patient comorbidities, a two-stage reconstruction may be the best treatment option. The first approach includes the Masquelet technique, the Papineau technique, or antibiotic-impregnated cement and temporary fixation techniques [5]. When the infection has been cleared and the site is healthy, reconstruction includes multiple options, such as autograft bone transport utilizing external fixation [6], vascularized fibular transfer [7], massive cancellous bone grafting with and without tissue transfer [8], and osteomyocutaneous flaps.

Recently, the advancement of technologies has led to improvements in both the management and treatment of severe bone loss and deformities. Above all, 3D printing allows for the customization of implants based on the anatomy of each patient. This technology uses a technique of selective laser melting of metals (SLM), allowing the creation of specific object deponing materials layer upon layer [9]. What is required for the 3D technique is the precise acquisition of images and their accurate analysis: it is important to study both ankles so that the healthy one, supposing that the ankles are symmetrical, can be used as a model for the other. Taking into consideration the ankle joint, the greatest difference observed in the volume of the entire talus was 7%, whereas the greatest difference in surface area observed was 5%; the difference was smaller if only the talar trochlea was considered. When considering the distal tibia, the difference was 3% for both parameters [10].

When the patient’s anatomy is outside the range of standard implants or when the patient suffers from severe bone loss or a severe deformity, custom-made implants are indicated [11].

This article presents a case series from the authors’ personal experience in treating wide post-infectious distal tibia/ankle bone loss using custom-made 3D-printed patient-specific spacers.

## 2. Materials and Methods

Informed consent was obtained from all patients before the surgery was scheduled, following the principles of the Declaration of Helsinki. All study data were treated with maximum confidentiality.

### 2.1. Planning Procedures and 3D Technology

For patient-specific analysis of orthopaedic applications, accurate medical imaging of the patient must be obtained. Modern multidetector computed tomography (MDCT) and magnetic resonance imaging (MRI) techniques provide fast and accurate 3D data with high resolution.

Converting digital medical images into a three-dimensional physical object involves three steps: image acquisition, digital image processing, and, lastly, 3D printing.

Image processing is based on a CT scan and completed with soft-tissue information provided by MRI. Digital image processing software (InVesalius 3, CTI, Campinas, Brazil) extracts DICOM images for data reconstruction. 

The subdivision of images into anatomical segments is necessary for the selection of anatomical regions of interest within the data. Computer-assisted design (CAD) software (InVesalius 3, CTI, Campinas, Brazil) transforms the contours of a 3D model into a series of triangles, the number of which directly correlates with the resolution. The CAD information is then converted into a stereolithography format (STL). Further editing of STL files can be performed before the CAD data are sent to a 3D printer for object fabrication. DICOM images are thus the key links between 3D printing technology and patient-specific image data.

The CAD software analyzes the STL file representing the 3D model to be manufactured and cuts the model into a series of layers. A 3D printer then fabricates the physical model in three dimensions by adding successive layers of material to recreate the virtual cross sections. The 3D printing technology used in medicine can be classified according to production techniques. The main ones are stereolithography (SLA), molten deposition modelling (FDM), selective laser sintering (SLS), and electron beam melting (EBM). In selective laser sintering, a laser beam is used to sinter dust particles of thermoplastic or metal. The implant used in this study was made of trabecular titanium and was manufactured by the Istituto Tecnològico de Canarias (Las Palmas de Gran Canaria, Canarias, Spain). The engineer was present at the time of the surgery in order to guide the surgical team to better use the specific instrumentation during the implant.

### 2.2. Study Population

This study included four patients (3 M and 1 W) aged between 55 and 64 years. Only one of them used to smoke, and none of them had diabetes. All the patients had a history of tibial pilon fracture (one of them exposed) with subsequent chronic osteomyelitis. The comorbidities and demographics of the study population have been resumed in Table 1.

All the patients underwent surgical procedures at the IRCCS Istituto Ortopedico Rizzoli (Bologna, Italy) between 2016 and 2020.

**Figure 1:**
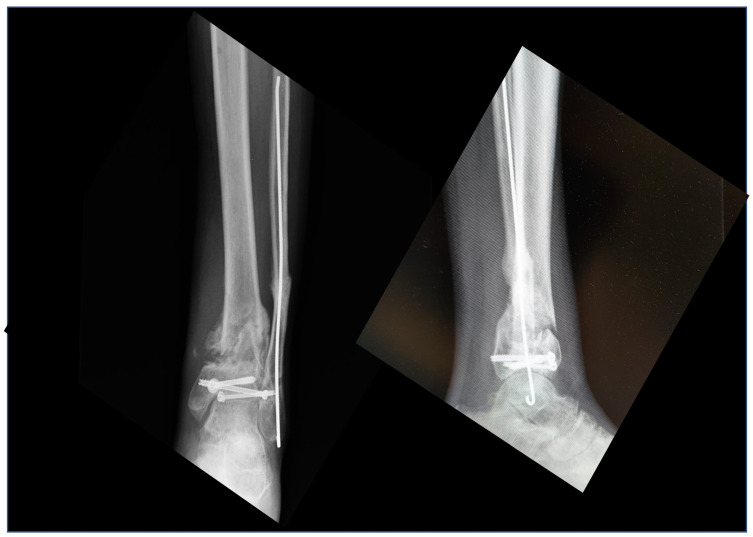
Pre-operative X-rays showing a case of septic non-union of the tibial pilon.

**Figure 2:**
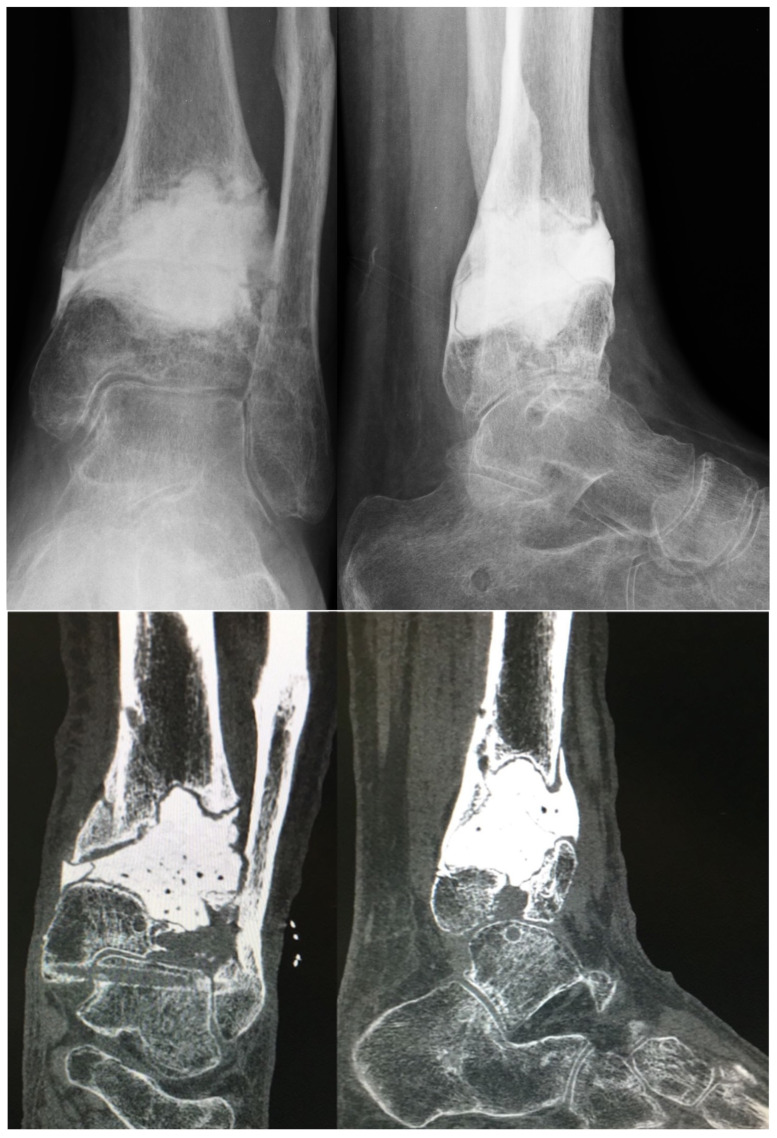
X-rays (**above**) and CT scan (**below**) of the previous case after wide surgical toilette, bone re-section, and positioning of an antibiotic-impregnated cement spacer.

The patients presented a clinical picture of tibial pilon septic non-union (Figure 1) confirmed by laboratory analysis (erythrocyte sedimentation rate—ESR and C-reactive protein—PCR, cultural and histological samples) and imaging (single-photon emission computerized tomography—SPECT and labelled leukocyte scintigraphy). Hardware removal and deep surgical toilette associated with a wide resection of the infected bone tissue were performed, followed by antibiotic cement spacer positioning (Figure 2). Based on the results of the microbiological tests, a targeted maximum performance antibiotic therapy was set up by infectiology consultants. The stabilization of the surgical site was performed by a linear external fixator. 

Analyzing the patients’ bone loss, a tibiotalocalcaneal (TTC) custom-made reconstruction/arthrodesis was considered. A comparative ankle CT scan was sent to the engineering service to produce a custom-made trabecular metal reconstruction spacer and its respective resection guides. The spacer was centrally drilled along the long axis to allow the construct to be stabilized with a retrograde intramedullary nail.

**Figure 3:**
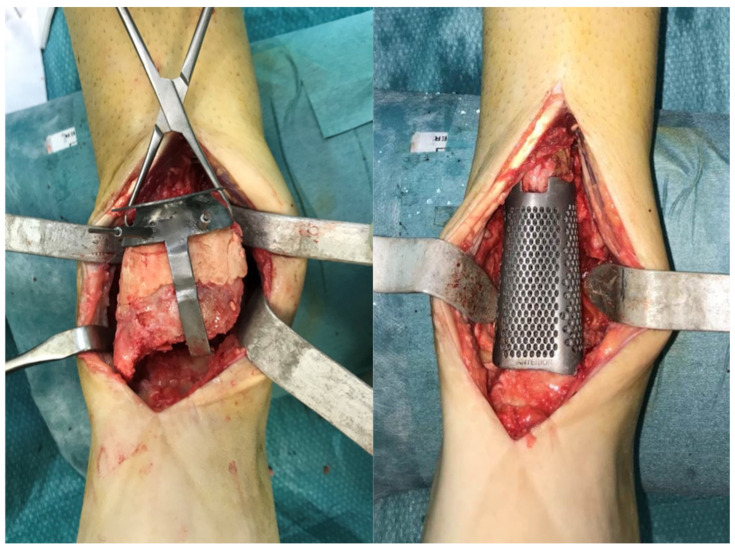
Intra-operative image. Guided resection using custom-made cutting guide (**left**) and implanted 3D-printed trabecular metal spacer (**right**).

**Figure 4:**
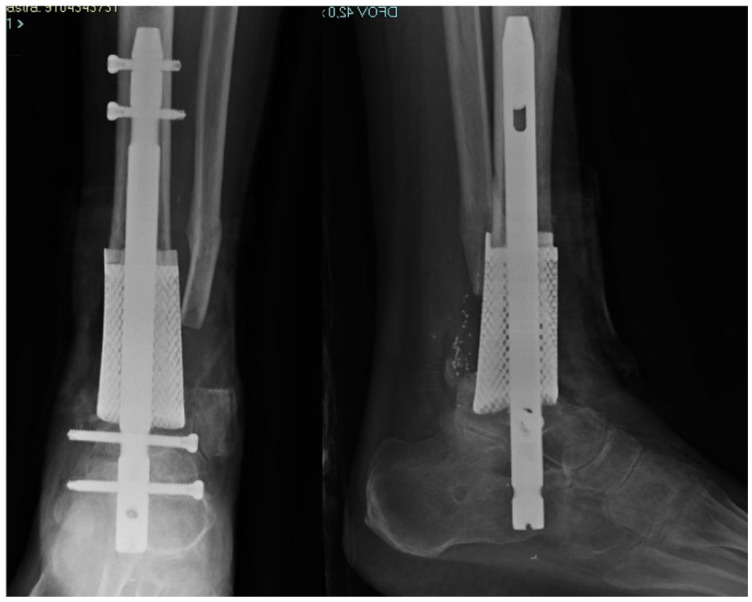
Post-operative X-ray of the final custom-made construct.

Once the infection was eradicated and this had been confirmed by laboratory, imaging, and clinical assessments, the patients underwent the main surgical procedure: through an anterior ankle approach, guided tibial resection, using a custom-made 3D-printed cutting guide to regularize the proximal and distal bone surfaces, positioning of the custom-made metallic spacer, and retrograde TTC nailing were completed (Figure 3 and Figure 4). The subtalar joint was not approached and prepared. Arthrodesis of the subtalar joint was achieved by exploiting the mechanical action of the nail and fibrosis following bleeding in the subtalar joint itself [12]. 

The clinical outcomes were evaluated in all patients through the validated AOFAS ankle–hindfoot score (American Foot and Ankle Society) [13] and the Visual Analogue Scale (VAS). Clinical outcomes were measured preoperatively and at the last follow-up. Patients who did not have at least one year of follow-up were excluded. Bony consolidation was evaluated both radiologically and clinically: radiologically, based on the FU X-rays that were examined by a dedicated radiologist (in particular, bone growth at the proximal and distal edges of the implant); clinically, by evaluation of the patients’ clinical scores and objective stability. 

## 3. Results

Four patients underwent tibiotalocalcaneal (TTC) custom-made reconstruction/arthrodesis. The mean age was 59.3 (range: 55–64; SD: 3.49); three patients were men, and one was female. All patients reported a minimum follow-up of 12 months, with a period between 18 and 45 months (mean: 29.50 months; SD: 11.50). 

Both validated AOFAS scores and VAS scores, at the last FUs, showed improvements compared with the pre-operative scores. Validated ankle–hindfoot AOFAS score improved from a pre-operative mean of 52.17 (range: 42–70; SD: 2.8) to a mean of 80.25 (79–89; SD: 1.8) and VAS score improved from an average of 6.75 (6–8; SD: 6.82) to an average of 0.8 (0–1; SD: 0.70).

No major complications have been registered; a wound healing delay has been reported in one patient, treated with advanced dressing and without signs of local infection.

**Figure 5:**
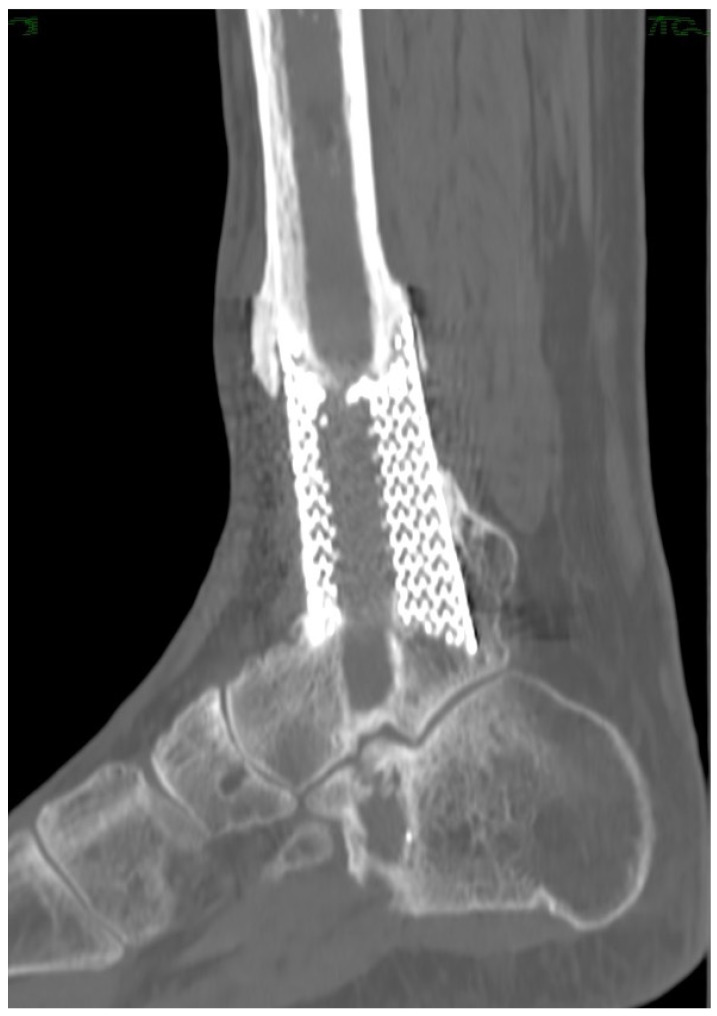
CT scan at final follow-up, after intramedullary nail removal. Note the proximal and distal osteointegration of the custom-made implant.

Radiological signs of osteointegration of the metallic spacer have been shown between 4 and 6 months in all patients. The nail was removed in one case because of intolerance, after verifying successful integration of the spacer (Figure 5).

## 4. Discussion

Historically, tibial plafond chronic osteomyelitis is one of the most challenging problems encountered by orthopaedic surgeons. Patient clinical history could be connected to a higher risk of developing chronic osteomyelitis; in particular, diabetes, as well as smoking, increases the risk of developing such complications. In addition, bone exposure at the time of the fracture increases the risk of osteomyelitis. None of our cases had diabetes, only one of them was a smoker, and only one had a history of exposed fracture. In past decades, distal tibial and ankle bone loss has been treated with arthrodesis and bone grafts because this treatment guarantees long-term pain control even though joint movement is sacrificed. As shown in this case series, extreme bone deformities and significant bone defects remain a good indication of ankle arthrodesis as well. Recently, there have been huge technical developments in the production techniques of 3D printing, extending the range of shapes, designs, and sizes of implants and reaching an unlimited level of customization that was previously inaccessible [14].

Before the use of custom-made implants was brought into clinical practice, the most used approaches in patients who suffered excessive bone loss from severe deformities included autograft bone transport utilizing external fixation and bone grafting. Compared to the more commonly used technique of bone transport with external fixation, no complications, including non-union, malalignment, or refracture at the regenerate site or pin tract infections, have been described using 3D-printed custom-made implants. Moreover, the non-weight bearing can be shorter, reducing the stress for the patient [14] and the use of autografts has the considerable disadvantages of donor site morbidity and limited available quantity [15,16,17]. Allografts are also potentially limited in size and are less osteogenic, with higher rates of non-union [18]. Both have been known to undergo late collapse and can be limited with respect to the ability to fully achieve the correct shape for reconstruction [19,20].

During the last follow-up, the pain and disability presented in the previous clinical pictures of the patients were reduced. The results obtained in these cases are not different from those retrievable in the literature, even though the mid-term follow-up makes it difficult to make any kind of prediction of outcomes in the long term [21]. Unfortunately, because of their recent development, there are no data on the average survival of custom-made 3D-printed implants. Even though it is easy for the authors to assume an increase in the lifespan of these implants compared with traditional devices because of their ideal adaptation to the patient’s anatomy, a long-term follow-up is needed to prove the thesis. Moreover, there are some concerns about the custom-made implants, such as the high costs, the longer time needed to design and produce the implant, the absence of intraoperative flexibility, and the technical difficulty of accurately positioning the implants. However, the specific details based on the anatomy of each patient achievable with 3D-printed designs cannot even be imagined with standard implants. Nonetheless, given the costs, it is extremely important to estimate the costs of the entire procedure, to evaluate the long-term possible clinical outcomes, and to choose very carefully the right patients [11].

Many factors can contribute to implant failure, for example, the unequal stress distribution at the bone–implant interface can cause micromovements of the prosthesis [14]. Conversely, custom-made prostheses may be the last or only possible option before definitive surgery, such as segment amputation. For a custom-made implant, good anchoring and primary bone healing is required, which is characterized by direct bone deposition at the bone–metal interface. In this case, the immediate stability of the implant and good compression between the implant and the bone are a prerequisite.

A factor worth discussing is certainly the lack of surgical preparation of the subtalar joint during surgery. As per surgical routine and the personal experience of the authors, TTC arthrodesis occurs by cartilage removal exclusively in the tibiotalar joint, this being a joint that normally has wide mobility, without the performance of a specific surgical step for the subtalar joint. This is because, in the opinion of the authors, the latter becomes rigid as a result of surgical trauma and bleeding, also following the removal of the endomedullary nail after the fusion of the tibiotalar joint, making, in the authors’ experience, the subtalar joint asymptomatic even without real “fusion”. In a previous case series, at long-term follow-up [12,22], complications such as rupture of the endomedullary implant or the need for surgical revision of the subtalar joint were not identified, but longer-term follow-ups will be needed to confirm this aspect.

The main limitation of this study is that the scientific relevance of the results obtained could be affected by the low number of patients included.

On the other hand, this aspect can be reasonably justified by the very definition of custom-made surgery, which is based on rare clinical scenarios and the use of patient-specific implants. 

## 5. Conclusions

Surgeries that, for anatomical reasons, were impossible to perform using standard implants are now achievable thanks to 3D-printed custom-made products. Moreover, the latest follow-up showed satisfying results, with significant pain reduction and better overall functionality in all patients. Although used with a small number of patients, 3D-printed implants have been demonstrated to avoid the complications and limitations of autografts and allografts in foot and ankle surgery. For these reasons, we consider, in selected patients with severe tibial pilon bone loss, reconstruction/arthrodesis with custom-made trabecular metal spacers a promising option.

However, longer-term studies are needed to monitor delayed complications, such as stress shielding and implant failure.

## Figures and Tables

**Table 1:** Comorbidities and demographics of study population.

	Sex	Age	Smoker	Comorbidity	Exposure
P1	F	55	No	Allergy to Rifamycin	Yes
P2	M	64	No	Hypertension, depression	No
P3	M	57	No	//	No
P4	M	61	Yes	Hypertension	No
